# DMARDs–Gut Microbiota Feedback: Implications in the Response to Therapy

**DOI:** 10.3390/biom10111479

**Published:** 2020-10-24

**Authors:** Oscar Zaragoza-García, Natividad Castro-Alarcón, Gloria Pérez-Rubio, Iris Paola Guzmán-Guzmán

**Affiliations:** 1 Faculty of Chemical-Biological Sciences, PhD program in Biomedical Sciences, Universidad Autónoma de Guerrero, Chilpancingo, Guerrero 39087, Mexico; zaragoza789@hotmail.com; 2 Faculty of Chemical-Biological Sciences, Universidad Autónoma de Guerrero, Chilpancingo, Guerrero 39087, Mexico; natycastro2@hotmail.com; 3 HLA Laboratory, Instituto Nacional de Enfermedades Respiratorias Ismael Cosío Villegas, Mexico City 14080, Mexico; glofos@yahoo.com.mx

**Keywords:** DMARDs, gut microbiota, dysbiosis, rheumatoid arthritis

## Abstract

Due to its immunomodulatory effects and the limitation in the radiological damage progression, disease-modifying antirheumatic drugs (DMARDs) work as first-line rheumatoid arthritis (RA) treatment. In recent years, numerous research projects have suggested that the metabolism of DMARDs could have a role in gut dysbiosis, which indicates that the microbiota variability could modify the employment of direct and indirect mechanisms in the response to treatment. The main objective of this review was to understand the gut microbiota bacterial variability in patients with RA, pre and post-treatment with DMARDs, and to identify the possible mechanisms through which microbiota can regulate the response to pharmacological therapy.

## 1. Introduction

The clinical practice guides for the treatment of rheumatoid arthritis (RA) establish different therapeutic schemes to diminish the clinical activity, limit the articular radiological damage progression, and the functional incapacity. However, response to treatment is variable and this effect can be attributed to genetic factors like polymorphisms in Human leukocyte antigen (HLA) genes or in genes that are involved in pharmacological metabolism pathways as well as clinical factors such as elevated antibody levels, cytokines and chemokines, symptom duration, and presence of comorbidities, among others [1,2].

Technological evolution has opened up the possibility of studying gut microbiota and also allows for the potential role in clinical characteristics and the response variability to RA treatment to be established. Originally, the microorganism recount in fecal culture allowed for the identification of big bacterial groups in the gut microbiota of RA patients [3,4,5,6]. The use of gas–liquid chromatography facilitated the identification of metabolites from bacteria [7,8] and real-time polymerase chain reaction (qPCR) analysis to quantitatively identify bacterial species [9,10,11,12,13]. Similarly, the analysis of bacterial 16s ribosomal ribonucleic acid sequencing (rRNA) [14,15,16,17,18,19,20,21,22,23,24,25,26,27,28,29,30,31,32] revolutionized the study of microbial diversity and the bacterial metagenome in RA [17,19,25,26,33,34,35,36,37,38]. In humans, the gut microbiome plays an important role in immunologic mechanisms and the inflammatory process. Changes in microbiota, influenced also by lifestyle and diet, may promote intestinal increased permeability and local inflammation, causing a spread of inflammation to the joints. Several nutrients such as polyunsaturated fatty acids, vitamin D, antioxidant, flavonoids, and probiotics present anti-inflammatory properties, featuring a protective role for RA development, while others such as red meat, high sugary drinks, and salt have a harmful effect [39]. Furthermore, the combination of probiotics and methotrexate (MTX) have been proven to contribute with the efficiency of the response to treatment [40,41] as well as in the specific variability of clinically relevant microbial species in the inflammatory process associated with RA [33].

Particularly, a great variability of bacterial species is shown in RA patients throughout the different clinical stages of the analyzed studies. However, cohort studies [6,25,33,37] allowed for an association between gut microbiota and pharmacological response variability in RA to be established. It is important then, to know the mechanisms that DMARDs put gut microbiota through and gut dysbiosis implications in the modulation of response to treatment as well as the strategies that should be followed to restore microbial symbiosis in RA.

## 2. Materials and Methods

A review of published English-based original research papers, reviews, and meta-analysis was conducted throughout the PubMed Central-NCBI search engines and the Web (from 1980 to May 2020), as well as in annual conference abstracts in rheumatology (American College of Rheumatology and the European League Against Rheumatism) were searched manually from 2013 to 2019. With regard to the researched papers that were set to review, Boolean operators “AND” and “OR” were considered as well as the terms “Microbiota”, “Microbiome”, “Gut microbiota”, “Rheumatoid arthritis”, “Early rheumatoid arthritis” “Arthritis”, “DMARDs”, “Drug-microbiota”, and “Response to treatment”.

**Figure 1:**
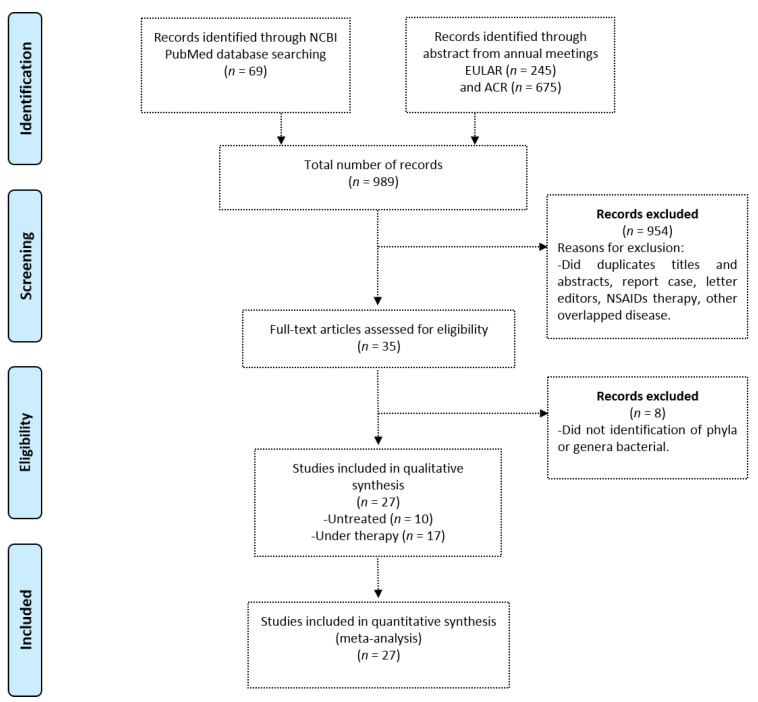
Flowchart illustrating the search results and selection process of journals included in this study, following the Transparent reporting of systematic reviews and meta-analyses (PRISMA) Statement.

The inclusion criteria were as follows: studies in patients with RA in pre-clinical phases, early RA, with or without pharmacological treatment regardless of the administered therapeutic schemes; and studies of in vivo and in vitro models; cross-sectional studies; case-control studies; and cohort studies. The review included studies based on conventional methodologies such as bacterial culture, analysis of qPCR, pyrosequencing, 16s rRNA, and bacterial metagenome (Figure 1).

## 3. Results

### 3.1. Disease-Modifying Antirheumatic Drugs (DMARDs) Usage in RA

International guides of clinical practice recommend the use of DMARDs as a pharmacological treatment in RA. These could have a conventional synthetic origin (csDMARDs) such as methotrexate (MTX), sulfasalazine (SSZ), or leflunomide (LEF) and could be administered in monotherapy or a combined therapy. They could also be administered along with the gradual and temporal use of corticosteroids (Cs) [42]. Hydroxychloroquine (HCQ) and chloroquine (CLQ) are also recommended drugs [43,44]. Recently, treat to target therapy (T2T), which includes the combined use of MTX + SSZ + HCQ [45], has been suggested as the best therapy because of its efficiency at accomplishing clinical remission in RA patients through the rational use of drugs and because it is economically feasible [46]. The use of biological DMARDs (boDMARDs) [42,47,48,49,50] or biosimilar DMARDs (bsDMARDs) is also included in treatment schemes when faced with a poor response to conventional drugs [51]. Nonetheless, the use of csDMARDs is still the principal strategy in RA treatment globally.

### 3.2. Gut Microbiota and csDMARDs’ Metabolism

In vivo and in vitro studies revealed that gut microbiota has a role in the metabolism of approximately 50 drugs [52]. Zimmermann et al. [53] evaluated in an in vitro study 76 bacterial strains in gut microbiota and the metabolism of 271 drugs. They demonstrated that each bacterial strain metabolized from 11 to 95 drugs and that 176 drugs presented substantial metabolic change through the reduction of the drugs’ active molecules by some bacterial strain, which would allow the suggestion that the bioavailability of DMARDs is subjected to bacterial metabolism.

MTX is the key drug in the T2T scheme. It is used orally or parenterally in RA treatment [54]. MTX’s metabolism occurs through three different pathways. (1) Metabolized by gut bacteria in 2,4-diamino-N(10)-methylpteroic acid (DAMPA); a metabolite that represents less than 5% of MTX administered doses [55]. It has been proven that carboxypeptidase-G2 (CPDG2), a bacterial enzyme, induces the hydrolysis of MTX and non-toxic metabolite production such as DAMPA and glutamate [56,57,58,59]. In an in vitro study, it was proven that the species *Pseudomonas* catalyzed the synthesis of glutamate thorough CPDG2 from MTX [60], by which gut bacteria, which already modulates the drugs’ active metabolite availability, can also possibly modulate its effects. (2) The second metabolic pathway occurs in the liver, where MTX bio-transforms into 7-OH-MTX [61]. This metabolite is considered an inhibitor of human dihydrofolate reductase enzyme (DHFR). Gut microbiota also includes the DHFR enzyme, so it can modulate the drug’s metabolism, and at the same time, the drug can modulate microbial metabolism, thus creating a strong relationship. (3) The third pathway happens through the intracellular conversion of MTX into polyglutamates. This pathway is considered the most important one given that it contains the principal mechanism of immunomodulation [62]. The principal cells and tissue where MTX is metabolized into polyglutamate derivates are: fibroblasts, myeloid precursors, keratinocytes, cortical and trabecular bone, and enterocytes [63], so homeostasis and intestinal barrier integrity are essential for the principal active form of MTX synthesis.

Gut microbiota is a key element in intestinal mucus’ homeostasis and, aside from the fact that it has a direct participation in MTX’s metabolism, it can indirectly regulate pharmacological metabolism through the maintenance of intestinal barrier integrity. It is known that microbial dysbiosis has an impact on mechanisms of translocation, immunomodulation, metabolism, and enzymatic degradation on a gut level and it compromises microbial diversity [64]. It was reported that administering high doses of MTX produces antibacterial activity, thus in vivo diminishing *Bacteroidetes* abundancy and the increase of *Firmicutes* [25,65]. Similarly, it was observed in murine models that treatment with MTX diminishes the abundance of *Bacteroides fragilis* [66], but not the one from the order *Lactobacillales* [67].

On the other hand, it was also proven that SSZ has effects over gut microbiota when it is administered in monotherapy or in combination with MTX [42,68]. SSZ is metabolized by gut microbiota through chemical reactions that are mediated by azoreductases, which reduce SSZ into sulfapyridine and 5-aminosalicylic acid (5-ASA/mesalazine). This last one is considered an anti-inflammatory component [52]. Most of 5-ASA is held in the colon and experiences enterohepatic re-circulation and finally is excreted in the feces [69]. At the same time, 5-ASA could be inactivated by microbial arylamine of N-acetyltransferases (NATs) [70], inhibiting the anti-inflammatory effects of the drug. Sulfapyridine, on the other hand, has anti-microbial effects [71] and is also metabolized in the liver through the acetylation by the arylamine NAT-2, hydroxylation, and glucuronidation [72,73]. Due to all of the above, the abundance of bacteria that produces azoreductases—*Bifidobacterium*, *Lactobacillus*, *Enterococcus*, *Clostridium*, *Eubacterium*, and *Bacteroides* genus [74,75,76], and bacteria that produces NATs could have a relevant impact on the markers that define the response to RA treatment.

Regarding the use of LEF, it was established that its mechanism of immunomodulatory action happens through its active metabolite A 77 1726, which participates in the inhibition of pyrimidine’s synthesis de novo by inhibiting dihydroorotate dehydrogenase and as a consequence, the lymphocyte proliferation [77,78]. To date, there is no direct evidence between LEF treatment and gut microbiota modulation, however, it is known that *Eggerthella lenta* uses ornithine as a substrate to generate energy, thus producing citrulline and carbamoyl-phosphate synthase. This last one is involved in the pyrimidine pathway, so its production by microbial species could be related to the active metabolism of LEF [78], and the production of citrulline along with the presence of citrullinated antigens of bacterial origin. Nonetheless, in the study of Chen et al. [19] the elevated abundance was not related to the citrulline serum levels.

CLQ and HCQ are drugs that are included in the treatment scheme for RA [43] because of their effects in the inhibition of the processing and presentation of antigens [79] and because they limit the activation and proliferation of T-lymphocytes and the synthesis of pro-inflammatory cytokines [80] such as TNF-α, IL-1β, IL-6 [81], IL-17, and IL-22 [82]. In a model of arthritic rats’ K/BxN, it was shown that the consumption of HCQ increased the intestinal abundance of *Akkermansia* and *Parabacteroides* while diminishing *Clostridium sensu stricto*-1 [83]. In RA patients, Chen et. al. [19] reported that the intake of HCQ increased the bacterial diversity. Recently, it was described that the T2T scheme modulated the presence of bloodstream bacteria in RA patients by promoting the abundance of the genera *Haemophilus, Alloprevotella, Eremococcus*, and *Lachnospiraceae_UCG001*, possibly translocated from classic niches of the human microbiome [31].

The effect of boDMARDs on gut microbiota has also been shown in an arthritic rat model DBA/IJ, treated with etanercept (ETN). It was reported that there was a decrease in the relative abundance of bacterial genus *Escherichia/Shigella* and the genera *Lactobacillus*, *Clostridium XVIa,* and *Tannerella* [84]. This only strengthens the evidence of the intimate relationship between gut microbiota and the immune system, given that boDMARDs can have a direct immunomodulatory effect, local or systemic, over their cellular targets as well as the indirect way through gut microbiota modulation.

### 3.3. Pre- and Post-Treatment Intestinal Dysbiosis with DMARDs in RA

The analysis made with avant-garde technologies has made it possible to identify the bacterial lineage expansion associated with the etiology, clinical progression, and possibly with the response to RA treatment.

#### 3.3.1. Firmicutes

The phylum *Firmicutes* is one of the most plentiful in gut microbiota including the classes *Bacilli, Mollicutes* and *Clostridia*. In pre-clinical phases of RA, it was reported that there was an increase in the abundance of the family *Lactobacillaceae* [22,24,30], a phenomenon also observed in patients with a clinical diagnosis of early RA. In these cases, it particularly reported the increase in *Lactobacillus salivarus, Lactobacillus iners*, and *Lactobacillus ruminis*, which has suggested that the changes in bacterial quantity and structure of *Lactobacillus* could be an associated factor to the development and the progression of RA [10].

In patients treated orally with MTX, an increase of the phylum *Firmicutes* in gut microbiota was reported [25]. Zhang et al. [33] reported that the use of MTX is associated with the restoration of gut microbiota because it favors the increase of *Lactobacillus salivarus*, *Lachnospiraceae bacterium* and *Clostridium asparagiforme*, and the decrease of some *Firmicutes* of the family *Veillonellaceae* of oral microbiota. However, in a particular manner, *Lactobacillus salivarus* was related to the presence of a larger clinical activity, so the recount of *Lactobacillus* could represent a gut microbiota restoration marker, subjected to the definition of the species’ proportion that forms the family *Lactobacillaceae*. Other studies have established the relation between bacterial species of the genus *Lactobacillus*—*Lactobacillus fermentum*, *Lactobacillus gasseri*, *Lactobacillus ruminis*, *Lactobacillus reuteri*, and *Lactobacillus plantarum*—and boDMARD treatment in RA patients for at least six months [9]. The same research group demonstrated that treatment with tocilizumab and infliximab in six months, could be possibly related to the decrease of *Lactobacillus plantarum* and *Lactobacillus gasseri*. Therefore, boDMARDs show an effect over the commensal decrease [11] and over important changes in the microbiome, which could be greater than the effects of using csDMARDs.

The abundance of microorganisms of the *Clostridia* order has been reported to increase in RA patients without pharmacological therapy [16], *Clostridium perfringes* [3] and *Clostridium III* [23] in particular. The presence of *Clostridium perfringes* alpha toxin is related to the RA autoimmunity process and an elevated clinical activity of the disease [3,5,85,86]. The immunosuppressor effect of boDMARDs has been tested, and in patients that were treated with ETN, the species of the family *Clostridiaceae* has been reported as diminished, *Clostridium coccoides* amongst them [11]. However, in patients who were in combined therapy of boDMARDs + csDMARDs, the effect over the family *Clostridiaceae* has been positive [34]. The same in patients who were only treated with MTX [37], hence the metabolites of csDMARDs appear to regulate the growth of bacterial groups of the *Clostridia* class in the gut. Other treatment schemes have been associated with other *Clostridial* species’ proportions. In patients with combined csDMARDs therapy, a decrease in *Clostridium leptum* was reported, meanwhile, in patients who were treated with csDMARDs + Cs, an increase was reported [27]. Consequently, it is important to analyze the effects of RA therapy over specific bacterial species given the different metabolic characteristics they have and their potential relationship with the clinical characteristics and therapy response.

In RA patients, the abundance of the family *Enterococcaceae* and the *Enterococcus* genus is reported recurringly. This increase has been described in RA patients without pharmacological treatment [16,30], in those treated with boDMARDs for at least six months [9,11], and in those in combined therapy of csDMARDs + boDMARDs [32]. *Enterococcus* does not appear to be directly related to RA treatment, but it does seem related to the development of septic RA in prosthetic joints [87] and with the preoperative infection of the knee in RA patients [88].

On the other hand, the abundance of the genus *Faecalibacterium* and the *Faecalibacterium prausnitzii* species was reported as decreased in RA patients without pharmacological therapy [22], whereas in patients who were treated with MTX, there was an increase [19]. Interestingly, in patients with other types of autoimmune diseases such as Sjögren Syndrome [89] and Juvenile Idiopathic Arthritis [90], the decrease in genus *Faecalibacterium* was also reported. *Faecalibacterium prausnitzii* is a commensal bacterium that produces butyrate in the colon and its intestinal abundance is considered a health bioindicator given that its decrease is associated with the presence of inflammatory processes [91,92]. Some of the effects of MTX treatment in RA patients could be related to the capacity of stimulating the abundance of *Faecalibacterium*, so the analysis of other butyrate producing genus and its immunomodulatory role associated with MTX treatment and other csDMARDs is most important.

The variability of bacterial genera and species that are related to RA treatment is very broad: while the *Faecalicoccus*, *Streptococcus*, and *Gemmiger* genera are abundant, *Rhoseburia*, *Sporobacter*, *Anaerofustis*, and *Lachnospira* diminish in RA patients without treatment [23]. *Eubacterium* is another genus that increases in patients who were treated with a combination of boDMARDs + csDMARDs [34], whereas *Dorea formicigenerans* decreases [32]. One of the most used therapies in RA treatment is csDMARDs + Cs, and it has been observed that this combination promotes the increase of *Rhutennibacterium lactatiformans*, *Anaerotruncus colihominis*, *Christensenella minuta*, *Dialister invisus*, and *Harryflintia acetispora* [27]. Nonetheless, the relation between each one and the response to DMARDs has not been described, which makes it important to study whether the bacterial metagenome of these genera and species is related to the metabolism of DMARDs as well as whether a microbial cluster exists that could be related with clinical prognosis markers and the response to RA treatment as well as the potential diet–drug interaction in the modulation of gut microbiota diversity in RA patients.

#### 3.3.2. Bacteroidetes

The phylum *Bacteroidetes* includes the *Bacteroides, Sphingobacteria, Cytophagia,* and *Flavobacteria* classes. The class *Bacteroidia* and the order *Bacteroidales* has been reported in greater abundance in RA patients without pharmacological therapy [26,28]. In an in vivo model, it was shown that *Bacteroides fragilis* polysaccharide A mediates the conversion of naive TCD4^+^ cells into Foxp3^+^ and CD39^+^ cells as well as the IL-10 secretion through the activation of the toll-like receptor 2 [93,94,95] and the decrease of cytokines that are linked to the Th17 profile [96]. Additionally, the derived butyrate of *Bacteroides fragilis* is associated with the differentiation of Treg cells and a tolerogenic state at the gut level [97]. The decrease in phylum *Bacteroidetes* related to pharmacological treatment could potentially compromise the immunoregulatory function and the presence of clinical manifestations in RA, along with the presence of other intrinsic and extrinsic factors in the patient such as advanced age, associated comorbidities, and gastrointestinal tract lesions. Likewise, the presence of *Bacteroides fragilis* causes pyogenic RA [98,99].

In patients that are in a preclinical stage of RA, an abundance of the family *Prevotellaceae* [24] has been observed whereas in patients with clinical RA, a decrease of the genus *Alloprevotella* [28] and *Prevotella* [14] has been reported as well as the species *Prevotella* spp. [24] and *Prevotella copri* [12,17,20]. In RA patients without pharmacological therapy, the abundance of *Prevotella copri* is as high as 75%, while in patients with chronic RA, the proportion was only 11.5% [17]. Maeda et al. [12] reported that a third of RA patients presented high levels of *Prevotella* and IgA antibodies against *Prevotella copri* as well as their relationship with Th1 and Th17 related cytokine and chemokine serum levels [100]. *Prevotella copri* is an obligate anaerobic microorganism and it has been suggested that it is internalized in phagocytic cells and joints due to the presence of the DNA of *Prevotella copri* detected in the synovial fluid of RA patients [100,101]. Moreover, ribosomal protein L23A in *Prevotella copri* has been linked with the stimulation of dendritic cells and with the synthesis of cytokine IL-17, thus contributing to the maintenance and exacerbation of the inflammatory process in RA [20]. *Prevotella copri* could produce phosphoadenosine phosphosulfate reductase, an oxidoreductase enzyme involved in sulfur’s metabolism, and in the synthesis of thioredoxin, which has been related with a greater clinical activity in RA by increasing oxidative stress [102]. In RA patients that are carriers of the shared epitope, a larger abundance of gut microbiota *Prevotella copri* has been observed [17]. The interaction between the microbiota and susceptibility genes for the development and clinical prognosis of disease contributes to the diversification of the clinal phenotype of RA.

In RA patients, it has been proven that the combined treatment of boDMARDs and MTX or with SSZ is associated with the decrease in families *Prevotellaceae* and *Paraprevotellaceae* in gut microbiota [21]. However, the sole therapy of csDMARDs has proven its relation with the increase in *Prevotella* spp.’s abundance [13], but also with the decrease in the proportion of *Prevotella/Bacteroides fragilis* [12]. The complexity in the analysis of microbial species that are possibly related to pharmacological metabolism indicates that the bacteria establish metabolic pathways that converge in mediating a key spot of pharmacological transformation, bioavailability, and absorption.

#### 3.3.3. Proteobacteria

*Proteobacteria* is one of the major bacterial phyla in gut microbiota. In RA patients without pharmacological therapy, a high abundance of genera *Colibacterias* [16] and *Escherichia-Shigella* has been reported [28]. However, in untreated patients as well as in those treated with MTX, a decrease of *Haemophilus* spp. [33] of genus *Enterobacter* [28] and the order *Enterobacteriales* [22] was observed, whereas treatment with boDMARDs favored the increase of *Enterobacteriaceae* [11], *Proteobacteria*, *Klebsiella*, *Desulfovibrionaceae*, *Succininivibrionaceae* [21], *Epsilonproteobacteria*, and *Campylobacteria* [34], and the decrease of the genus *Deltaproteobacteria* [22].

#### 3.3.4. Actinobacteria

Species diversity of phylum *Actinobacteria* is also wide in RA patients. Forbes et al. [23] reported an increase of genera *Actinomyces*, *Eggerthella*, and *Rhotia* in the gut microbiota of RA patients without pharmacological therapy. Nevertheless, the decrease in genus *Collinsella* [26] suggests a negative relation with the inflammatory process in RA. In a model of HLA-DQ8 arthritic rats, the presence of *Collinsella aerofaciens* was associated with the accelerated progression of RA. This effect is related to metabolite production such as alfa-aminoadipic acid and asparagine, which promotes IL-17A production and the increase in intestinal permeability by diminishing the expression of tight junction protein zonula occludens-1 (ZO-1), thus contributing with systemic inflammation [19] and with metabolic endotoxemia.

Combined treatment of MTX + HCQ in RA patients has not evidenced a relation with the variability of *Eggerthella lenta* and *Collinsella aerofaciens* [19], although MTX treatment favors the increase of *Gordonibacter pamelaeae* and *Eggerthella lenta* in the oral microbiota of RA patients [33]. As for boDMARDs, they could have a larger effect on phylum *Actinobacteria*, as shown in Chiang et al.’s study [29], particularly over the sequence number of *Collinsella aerofaciens* [32] and the decrease in genus *Bifidobacterium* [11]. At the same time, the csDMARDs + boDMARDs + Cs combination affects the decrease of the family *Bifidobacteriaceae* [21].

#### 3.3.5. Other Bacterial Phyla

Treatment with combined therapies of csDMARDs + boDMARDs has shown an effect on the abundance of genera *Akkermansia* [29], *Tenericutes*, and *Sinergistetes* [21] as well as an increase of *Cyanobacteria* and *Nostocales* in patients treated with ETN [22]. The abundance of other bacterial phyla in future studies could reveal their functions in the response to RA treatment.

**Figure 2:**
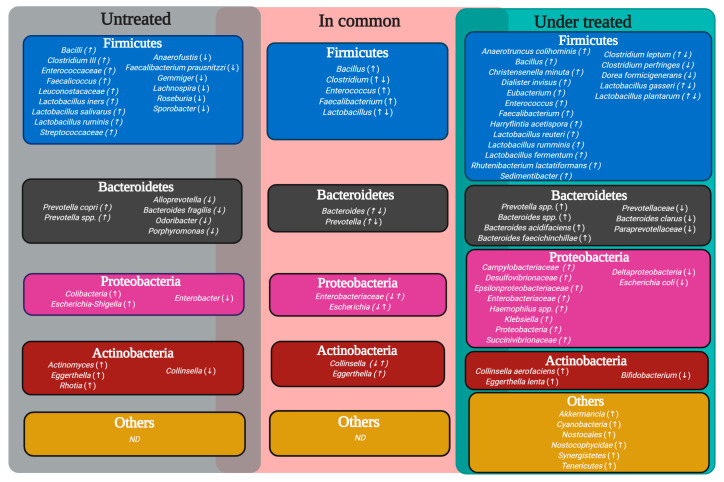
Variability in the gut microbiota in RA under treated and untreated with DMARDs. Genera colors represent phylum and ↑/↓, increased/decreased status in patients with rheumatoid arthritis untreated or under treated. DMARDs, disease-modifying antirheumatic drugs; RA, rheumatoid arthritis; ND, non-describable.

In this regard, when pre- and post-DMARDs treatment studies have been compared, it was shown that several bacterial genera and species were preserved in similar proportions between groups, while others varied significantly (Table 1 and Table 2). Additionally, the pharmacological combination could promote an important degree of dysbiosis by affecting the gut microbiota’s most representative bacterial phyla (Figure 2).

### 3.4. Gut Microbiota and Its Relation with the Response to Pharmacological Treatment

**Figure 3:**
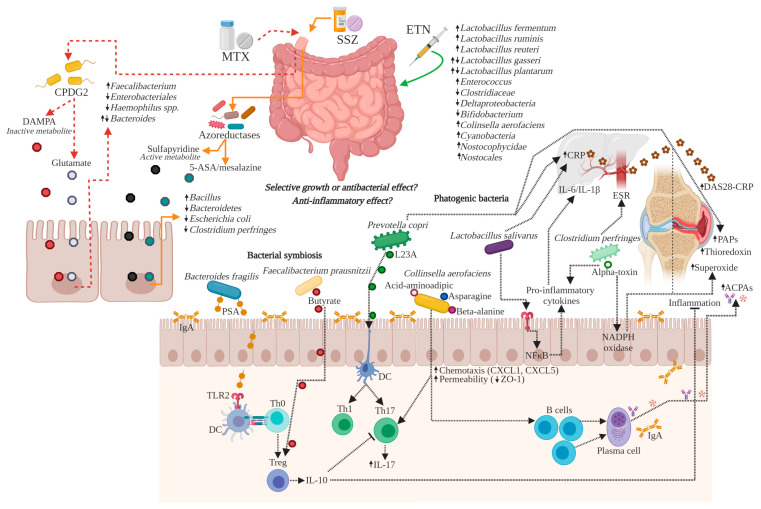
DMARDs-gut microbiota feedback in RA. Interactions of the MTX, SSZ, and ETN on the gut microbiota with effects direct on selective bacterial growth and antimicrobial effect. ↑/↓ = increased/decreased. ACPAs, anti-citrullinated peptide antibody; CXCL, chemokine ligand; ***CPDG2***, carboxypeptidase-G2; CRP, C-reactive protein; DAMPA, 2,4-diamino-N(10)-methylpteroic acid; ***DC***, dendritic cell; DMARDs, disease-modifying antirheumatic drugs; DAS28, disease activity score 28; ESR, erythrocyte sedimentation rate; ETN, etanercept; IgA, immunoglobulin A; IL, interleukin; L23A, ribosomal protein L23A; MTX, methotrexate; NADPH, nicotinamide adenine dinucleotide phosphate; NF-ĸB, nuclear factor kappa-light-chain-enhancer of activated B cells; PAPs, phosphoadenosine phosphosulfate reductase; PSA, polysaccharide A; RA, rheumatoid arthritis; SSZ, sulfasalazine; Th, T helper cell; TLR, toll-like receptor; Treg, regulatory T cell; ZO-1, zonula occludens-1. Created with BioRender.com.

As previously stated, it has been proven that treatment with DMARDs modulates gut microbiota. It has also been revealed that there is a relation of close feedback between DMARDs and possibly with its effects, an approximation is suggested in Figure 3. Particularly, it was observed that pharmacological treatment in RA increased the phyla *Firmicutes* [25,31] and *Bacteroidetes* [17,31]. This could be considered as a positive effect. However, the thin line that separates it from dysbiosis could depend on the intrinsic capacity to metabolize a drug-type xenobiotic or on factors such as the pharmacological combination, the administered dose, or the period the drug is prescribed. This consideration is to be discussed in favor of designing the best strategies in RA treatment.

A cohort study made by Neuman et al. [4] revealed an increase of *Clostridium perfringes* in the gut microbiota of RA patients that were non-responsive to pharmacological therapy. In addition to this, the presence of dyspepsia, oral and genital ulcers, hematomas, nausea, and dyspnea was also observed in post-treatment with SSZ [6], meanwhile, in responsive patients, *Clostridium perfringes* decreased [4,6]. Therefore, microbiota not only affects the clinical response in RA, but also in the adverse and toxic events that are associated to treatment intake, which increases the morbidity and mortality of the patient. Similarly, Kanerud et al. [103] reported that the use of SSZ is related to the decrease of *Escherichia coli* and *Bacteroides*, assigning to this csDMARD an antibacterial effect. Likewise, Isaac et al. [37] reported that MTX consumption favored the increase of the class *Clostridia* and the decrease in the class *Bacteroidia*, which in turn are related to the nonresponse to RA therapy. However, in another study, the increase of the phylum *Bacteroidetes* has been linked with the nonresponse to MTX treatment [25] (Table 3), so the decrease in the class *Bacteroidia* and the increase in genus *Bacteroides* [13,17,27] are related with treatment failure probability. It is important to analyze the biological characteristics related to the species’ variability or the bacterial proportions such as age, sex, diet, race, and even the clinical characteristics of RA patients, among others. Moreover, it has been proven that *Bacteroides* harbor antimicrobial resistance genes [104,105], which shows the possibility that some *Bacteroides* species are more sensitive or resistant to csDMARDs.

Chiang et al. [29] analyzed the gut microbiota in patients with different clinical phenotypes of RA and observed a low microbial α-diversity in patients with positive anti-CCPs antibodies, reporting a larger abundance of the genera *Blautia*, *Akkermansia*, and *Clostridiales*. Similarly, Chen et al. [19] reported a low microbial α-diversity in patients with elevated levels of rheumatoid factor, a clinical characteristic that Chen and colleagues [29] related to the increase in the proportion of *Blautia* and *Collinsella*. The study of *Collinsella*’s metabolome revealed that it produces three metabolites (beta-alanine, alpha-amino acid, and asparagine) that could be related to serological markers in RA. Particularly, *Collinsella aerofaciens* increases intestinal permeability by diminishing the expression of ZO-1, neutrophil chemotaxis, and IL-17A levels as well as the early development of RA in an in vivo model [19]. It was recently described that age is related in a negative manner to the *Collinsella aerofaciens* sequences, so its abundance in young patients could be representative. Additionally, it was observed that smoking habits and the elevated levels of anti-citrullinated protein antibodies have an effect on the abundance of *Collinsella aerofaciens*, which is why their presence could be implied in early stages of the disease and could represent a related factor with its expansion in RA. On a functional level, a connection was found between genes and the arginine deiminase enzyme [32], which means it could have an important role in the early stages of RA development. Due to this, a hypothesis arises which suggests that some gut bacteria could be involved in protein citrullination and with the autoimmunity level in RA. Bennike et al. [106] identified 21 citrullinated peptides in the colon tissues of RA patients, therefore the loss of intestinal integrity and the colon components that establish a tolerance site and the presence of citrullinated antigens could initiate and preserve immune activation processes in RA. The microbial profile identification establishes its potential use as bioindicators of clinical prognosis in RA and the development of supplementing strategies with probiotics or prebiotics to maintain microbial symbiosis and the metabolic processes associated with the metabolism of DMARDs.

Other potential mechanisms by which the microorganism abundance relates to the response to treatment are associated with the presence of the HLA-DRB1 allele. In a model with transgenic rat HLA-DRB1*0401, a higher abundance of genus *Clostridium* was found as well as a higher intestinal permeability and the synthesis of Th17 profile cytokines [107]. In humans, the positive reaction to the HLA-DRB1 alleles is associated with the presence of *Lachnospiraceae*, *Clostridiaceae*, *Bifidobacterium longum*, and *Ruminococcus gnavus* [108]. Another factor in the variability of response to treatment and microbiota is sex. It has been reported that the genera *Rikenellaceae*, *Porphyromonadaceae*, and *Coriobacteriaceae* are more abundant in women, whereas the genera *Pasteurellaceae*, *Butyricicoccus*, *Clostridiaceae 1*, *costridium sensu estricto 1*, and *Alisnonella* are more abundant in men [28]. All of this suggests that potentially, other mechanisms that are associated with sexual dimorphism and that interact with the microbiome could determine the response to treatment in RA.

The response variability to DMARDs relates to the presence of bacterial enzymes that are involved in their metabolism. It has been suggested that MTX could join equivalent bacterial DHFR such as *Escherichia coli* and *Lactobacillus casei* [109,110,111], and that the overexposure of a protein with DHFR activity reverts sensibility to intracellular MTX in strains of *Escherichia coli* that are resistant to the drug. This suggests that MTX is accumulated in cells where mutations of acrA or tolC have inactivated the resistance efflux pump to multiple AcrAB drugs, which depend on tolC [112] as well as that the gut bacteria can metabolize MTX into MTX polyglutamate [65]. This is possible when the biosynthetic pathway of tetrahydrofolate reductase, codified by intestinal metagenome, *Bacteroides* for example, can compete with the host’s DHFR and MTX’s metabolism, leading to only some of the patients to respond to the first scheme of the orally administered therapy [17] by intervening in the capacity of absorption and bioavailability of the drug inside the organism, modulating the anti-inflammatory effects of MTX.

Scher et al. [17] evaluated the metagenome of 14 RA patients without previous pharmacological treatment and five healthy controls. Interestingly, the metagenome of *Prevotella* is related to the metabolism of vitamins (biotin, pyridoxine and folate) and the pentose phosphate pathway. Similarly, Isaac et al. [35] analyzed 27 patients without pharmacological therapy and reported bacterial taxa and genes that were related to the metabolism of purines and MTX. They particularly described eight microorganisms that were associated with the modulation of 23 metabolic pathways and that MTX’s bioavailability was related to the scale of clinical response, thus suggesting a direct effect of gut microbiota on the response to therapy with MTX. On the other hand, Kishiwaka et al. [38] reported that the abundance of a gene related to redox reactions (R6FCZ7) increased in nine RA patients. The presence and abundance of species of the genus *Prevotella* could have an important role in the preservation of a redox environment, in the transport alteration and the metabolism of iron, sulfur, zinc, and arginine in the gut microbiota of RA patients [33]. These show that MTX could have a role in the metabolic modulation of commensal bacteria, thus contributing to the regulation of other bacteria or pathways that are related to nutrition, transport, and bacterial secretion processes.

## 4. Analysis and Future Perspectives

Bacterial genera *Lactobacillus*, *Clostridium*, *Prevotella*, *Collinsella*, *Faecalibacterium*, and *Eubacterium* are microorganisms associated with the presence of markers of clinical activity in RA, which is why studying them during different moments of the clinical course of the disease will help to understand their role in RA physiopathology and the response to treatment with DMARDs. In this review, the findings showed the presence of dysbiosis, mainly of the phyla *Bacteroidetes*, *Firmicutes*, and *Proteobacteria*, related to treatment with DMARDs. Dysbiosis, which is defined by the presence or by the relative abundance of microbial species like *Clostriduim perfringes*, *Lactobacilus salivarus*, *Collinsella aerofaciens*, *Faecalobacterium prautnizzi*, *Bacteroides fragilis*, and *Prevotella copri* establishes a close relation with gut-level immunomodulation processes and their systemic repercussion in the evaluation of the clinical response in RA.

This review presents a wide diversity of studies and reported microorganisms in RA populations from four continents. Although the variability of the results could be subjected to the analysis capacity and the detection of bacterial species through the use of different technologies, not every study included the analysis of the same group of hypervariable regions of 16s rRNA. In this regard, the evaluated regions in this review were the following: V1–V2 [17], V1–V3 [29], V2–V3 [32], V3–V4 [21,22,26,27,28], V3–V5 [19], and V4 [23,24,25,31]. Other techniques used in the analysis of prognosis with bioinformatic tools to evaluate associated genes to pharmacological metabolism and immunomodulation processes, could allow for a comprehensive analysis of the metagenome and the metabolome related with the response to DMARDs, or even with other analgesic-type drugs that are frequently used to limit the joint pain in RA, which have also been related with dysbiosis processes of gut microbiota through the interaction of nonsteroidal anti-inflammatory drugs (NSAIDs) with cell membrane phopholipids and the disengagement of mitochondrial oxidative phosphorylation as well as the increase in the production of free radicals, which leads to the increase in permeability and chronic intestinal inflammation [113,114,115]. It has also been reported that NSAIDs favor the decrease in bacteria from the genus *Lactobacillus*, an important group in the preservation of luminal pH, the permeability of intestinal mucosa, enterocyte adhesion, mucus production, and of immunomodulation processes [116,117]. In RA patients, it has been shown that the NSAID intake promotes the increase of bacteria from the genus *Eubacterium* and of cell wall fragments of *Eubacterium aerofaciens* [118] and *Clostridium* [5] as well as the decrease in the group *Bacteroides-Prophyromonas-Prevotella*, and *Eubacterium rectale-Clostridium coccoides* [15]. However, the combined treatment of DMARDs, corticosteroids, and NSAIDs has shown variable effects on microbiota [21].

The studies that were included in this review show geographic and racial variability, in addition to other characteristics that have a potential relation to intestinal dysbiosis in RA. A clear example of this is diet. Rodrigues et al. [13] evaluated the effect of diet over gut microbiota in Brazilian patients with RA. This study found a positive correlation (r = 0.26, *p* = 0.04) between animal protein consumption and the relative units of *Prevotella.* The dairy intake was correlated with the relative units of *Bacteroides* (r = 0.27, *p* = 0.04), the trans-unsaturated fatty acid consumption was related with the relative units of *Bifidobacterium* (r = 0.30, *p* = 0.02) and *Roseburia* (r = 0.26, *p* = 0.04), and the intake of hot beverages was related to the relative units of *Bifidobacterium* (r = 0.28, *p* = 0.03), *Roseburia* (r = 0.29, *p* = 0.03), and *Clostridium leptum* (r = 0.28, *p* = 0.03). It has also been observed that the consumption of vegetable-based diets and the increase of short-chain fatty acids favors the abundance of *Prevotella* and some *Firmicutes* [119]. Additionally, enterotypes that are associated with long-term diets of animal proteins and fat promote the abundance of *Bacteroides,* whereas other sugar-based diets increase the abundance of *Prevotella* [120]. Dietary palmitic acid could stimulate the growth of some *Bacteroides* such as *Bacteroides fragilis* and *Bacteroides thetaiotaomicron* [121].

It has been reported that supplementing the diet with fiber in RA patients could promote the increase of circulating Treg cell numbers and the decrease of bone erosion markers. The use of diets with high levels of fiber could be a complement to pharmacological strategies in RA treatment [122], additionally, a well-balanced food intake favors a greater diversity of intestinal microbiome and reduces metabolic and inflammatory risks [123]. It is suggested to establish a lifestyle modification program for RA patients based on three components: (i) low sodium and fat levels; a rich Mediterranean diet in fruits, vegetables, whole grains, and nuts, low on sugary drinks, red meat, processed meat, trans fatty acids; and supplementing with omega-3 fatty acids, non-essential amino acids and probiotics; (ii) physical activity according to each lifestyle; aerobic exercise and resistance training; and (iii) appropriate sleep hygiene and stopping smoking habits. This last scheme has positive effects in terms of limiting the progression of the disease [124]. It has been described that the intake of an anti-inflammatory diet is associated with a decrease in clinical activity (DAS28-ESR) in RA patients [125]. However, even though the effect related to the implementation of limited or cyclical fasting, vegan diets, Mediterranean, or dairy and gluten elimination has an important role in the decrease of clinical activity, and they present a hindrance in the possibility of keeping the diet long-term [126]. Diet modulation is also related to the use of alternative medicine. In one study, it was seen that *Tripterygium wilfordii* affects the decrease in *Holdemania filiformis* and *Bacteroides* spp., while the combination of *Tripterygium wilfordii* and MTX increased the abundance of *Prevotella intermedia* [33]. Nonetheless, available evidence still does not allow for the establishment of dietary interventions associated with the pharmacological scheme.

Many studies have demonstrated that dietary polyphenols modulate the composition and function of gut microbiota, interfering with bacterial quorum-sensing, membrane permeability as well as the bacteria’s sensitization to xenobiotics. Additionally, polyphenols can intervene in the intestinal metabolism and immunity because of the anti-inflammatory properties they have. The evidence from several studies in health and disease processes suggest that polyphenols express prebiotic properties and anti-microbial properties against pathogen microorganisms. Unfortunately, the therapeutic/nutraceutical use of polyphenols has been seriously compromised by low bioavailability, a property that limits reaching the objectives [127].

Moreover, the use of probiotics in RA has proven to modulate the clinical course of the disease. In a follow-up study two months after in RA patients, it was shown that the use of *Lactobacillus acidophilus*, *Lactobacillus casei*, and *Bifidobacterium bifidum* decreased clinical activity parameters, high sensitivity C-reactive protein (hsCRP) levels, insulin levels, and the HOMA-B index [128]. Other follow-up studies after two and three months reported that the administration of *Lactobacillus casei* 01 decreased hsCRP levels, the quantity of painful and inflamed joints, the global health scale, score DAS-28, and the levels of TNF-α and IL-12 [129,130]. In a general sense, a meta-analysis study reported that the administration of probiotics was associated with the decrease in IL-6 [131], CRP reduction, and a significant improvement in DAS-28 score [132]. The effect of intervention with probiotics and RA’s clinical activity markers have been shown in different animal models: in arthritic rats, it was reported that supplementing with *Lactobacillus casei* (ATCC334) favored the decrease of clinical activity markers, joint destruction, and of pro-inflammatory cytokines (IFN-γ, TNF-α, IL-1β, IL-17, and IL-6). It was also reported that the microbiota restoration with probiotics also favored the increase of *Lactobacillus acidophilus* and decreased the abundance of *Lactobacillus hominis*, *Lactobacillus reuteri*, and *Lactobacillus vaginalis* [133]. In the same way, the use of *Lactobacillus fermentum* PC1 in DBA/1 rats revealed that the levels of IL-4 and IL-10 increased, while synovial infiltration and cartilage damage decreased [134]. Another study in a Wistar rat model reported that probiotics affect the diminishing of inflammatory markers through the inhibition of intracellular signaling pathways and cytokine production [135]. In HLA-DQ8 arthritic rats, the use of *Prevotella histicola* has been reported to be related to the decrease in the Th17 response, a greater number of Treg cells, greater IL-10 transcription, and the decrease in intestinal permeability, thus limiting the severity of RA [136]. The former suggests that RA therapy must include csDMARDs + Cs + probiotics due to their effects in clinical improvement and the diminishing of associated comorbidities [129,130]. However, the probiotic choice is not an easy task given that even though different probiotics and prebiotics are known, suggested evidence in animal models must be tested in human clinical trials. In a Lewis rat model, the administration of combined MTX with *Enterococcus faecium* enriched with organic selenium decreased inflammatory markers with greater success than MTX monotherapy [40]. In another study with C57BL/6J rats, it was shown that caloric restriction for a short period and supplementing with *Lactobacillus rhamnosus* GG (ATCC53103) reduces the risks of intestinal damage during the administration of high doses of MT [67], hence limiting the adverse and toxic effects that are related to this treatment.

## 5. Conclusions

The extensive variability of the analyzed studies—in methodology, used tools in microbial analysis, sample sizes, participants’ ethnic origin, the disease’s clinical characteristics, doses, and studied pharmacological schemes—limit the possibility of establishing a concrete overview of microbiota in RA. Nevertheless, it does allow us to identify potential microbial profiles that are associated with the metabolism of DMARD and the possible mechanisms through which microbiota intervene in a failed response to therapy as well as to reveal the feedback that exists between microbiota-xenobiotics and the response of RA patients to therapy. Likewise, it would also allow us to implement strategies for microbial restoration, improve the response to DMARDs, and diminish the disease’s clinical activity markers and the adverse effects that are associated with practiced polypharmacy and its effects in non-responsive patients.

## Figures and Tables

**Table 1:** Studies of gut microbiota composition in untreated rheumatoid arthritis (RA) patients.

References	Country	Phylum			
		*Firmicutes*	*Bacteroidetes*	*Proteobacteria*	*Actinobacteria*
Maeda et al. [20]	Japan	NA	*Prevotella copri* (↑), *Bacteroides* (↓)	NA	NA
Maeda et al. [12]	Japan	NA	*Prevotella* (↑)	NA	NA
Jeong et al. [26]	Korea	NA	*Bacteroidetes* (↑) [*p* = 0.011], *Bacteroidia* (↑) [*p* = 0.014], *Bacteroidales* (↑) [*p* = 0.014]	NA	*Collinsella* (↓) [*p* = 0.004]
Liu et al. [10]	China	*Lactobacilli* (↑) [*Lactobacillus salivarus* (↑), *Lactobacillus iners* (↑), *Lactobacillus ruminis* (↑)]	NA	NA	NA
Sun et al. [28]	China	*Lactobacillus* (↓) [*p* < 0.001]	*Bacteroides* (↑) [*p* = 0.022], *Alloprevotella* (↓) [*p* < 0.001], *Odoribacter* (↓) [*p* < 0.001]	*Escherichia-Shigella* (↑) [*p* = 0.035], *Enterobacter* (↓) [*p* < 0.001]	NA
Tong et al. [30]	China	*Streptococcaceae* (↑) [*p* < 0.001], *Lactobacillaceae* (↑) [*p* < 0.001], *Enterococcaceae* (↑) [*p* = 0.029], *Leuconostacaceae* (↑) [*p* < 0.001]	*Bacteroidaceae* (↓) [*p* = 0.011]	NA	NA
Gul’neva and Noskov, [16]	Russia	*Enterococci* (↑), *Clostridia* (↑), *Lactobacteria* (↓)	NA	*Colibacteria* (↑)	NA
Toivanen et al. [14]	Finland	NA	*Prevotella* (↓), *Porphyromonas* (↓), *Bacteroides* (↓) [*p* < 0.001], *Bacteroides fragilis* (↓) [*p* < 0.001]	NA	NA
Alpizar-Rodríguez et al. [24]	Sweden	*Lactobacillaceae* (↑) [*p* = 0.039]	*Prevotella* spp. (↑) [*p* = 0.04]	NA	NA
Picchianti-Diamanti et al. [22]	Italy	*Bacilli* (↑) [*p* = 0.035], *Lactobacillaceae* (↑) [*p* = 0.021], *Faecalibacterium* (↓) [*p* = 0.012], *Faecalibacterium prausnitzii* (↓) [*p* = 0.006]	NA	NA	NA
Forbes et al. [23]	Canada	*Clostridium III* (↑), *Faecalicoccus* (↑), *Streptococcus* (↑), *Gemmiger* (↓), *Lachnospira* (↓), *Roseburia* (↓), *Sporobacter* (↓), *Anaerofustis* (↓) [*p* < 0.001],	NA	NA	*Actinomyces* (↑), *Eggerthella* (↑), *Rhotia* (↑) [*p* < 0.001]
Scher et al. [17]	USA	*Clostridia* (↓), *Lachnospiraceae* (↓)	*Prevotella copri* (↑), *Bacteroides* (↓)	NA	NA

↑/↓ = increased/decreased in RA patients without regimen to treatment. *p*-value < 0.05 for the association in the increase or decrease in the group of the phylum bacteria in the RA population. Legend: NA: not available; RA: rheumatoid arthritis.

**Table 2:** Studies of gut microbiota composition in under therapy RA patients.

References	Country	Phylum				
		*Firmicutes*	*Bacteroidetes*	*Proteobacteria*	*Actinobacteria*	Anothers
Maeda et al. [9]	Japan	*Lactobacillus fermetum* (↑) [*p* < 0.01] ^a^, *Lactobacillus gasseri* (↑) [*p* < 0.01] ^a^, *Lactobacillus ruminis* (↑) [*p* < 0.05] ^a^, *Lactobacillus reuteri* (↑) [*p* < 0.05] ^a^, *Lactobacillus plantarum* (↑) [*p* < 0.05] ^a^, *Enterococcus* (↑) [*p* < 0.01] ^a^	NA	NA	NA	NA
Maeda et al. [11]	Japan	*Lactobacillus fermetum* (↑) ^a^, *Lactobacillus gasseri* (↑) ^a^, *Lactobacillus reuteri* (↑) ^a^, *Enterococcus* (↑) ^a^ *Clostridium* del grupo c*occoides* (↓) [*p* = 0.003] ^a^, Subgroup *Lactobacillus gasseri* (↓) [*p* = 0.006] ^a^, Subgroup *Lactobacillus plantarum* (↓) [*p* = 0.017] ^a^	NA	*Enterobacteriaceae* (↑) ^a^	*Bifidobacterium* (↓) [*p* = 0.027] ^a^	NA
Lee et al. [27]	Korea	*Clostridium leptum* (↑) [*p* = 0.003] ^b^, Ruthenibacterium lactatiformans (↑) [*p* = 0.004] ^b^, *Anerotruncus colihominis* (↑) [*p* = 0.004] ^b^, *Christensenella minuta* (↑) [*p* = 0.024] ^b^, *Dialister invisus* (↑) [*p* = 0.030] ^b^, *Harryflintia acetispora* (↑) [*p* = 0.045] ^b^	*Bacteroidetes* (↑) [*p* = 0.034] ^b^ *Bacteroides acidifaciens* (↑) [*p* = 0.012] ^b^, *Bacteroides faecichinchillae* (↑) [*p* = 0.013] ^b^, *Bacteroides clarus* (↓) [*p* = 0.044] ^b^	NA	NA	NA
Zhang et al. [33]	China	NA	NA	*Haemophilus* spp.(↓) ^c^	NA	NA
Chiang et al. [29]	China	NA	NA	NA	*Collinsella* (↑) ^d^	*Verrucomicrobiae* (↑) [*p* < 0.05] ^d^, *Akkermancia* (↑) ^d^
Neumann et al. [4]	England	*Clostridium perfringes* (↓) [*p* < 0.05] ^e^	NA	*Escherichia coli* (↓) [*p* < 0.05] ^e^	NA	NA
Bradley et al. [6]	England	*Clostridium perfringes* (↓) [*p* < 0.05] ^e^	NA	*NA*	NA	NA
Kanerud et al. [103]	Sweden	*Bacillus* (↑) [*p* < 0.05] ^e^	*Bacteroides* (↓) [*p* < 0.05] ^e^	*Escherichia coli* (↓) [*p* < 0.05] ^e^	NA	NA
Breban et al. [21]	France		*Prevotellaceae* (↓) ^d^, *Paraprevotallaceae* (↓) ^d^	*Proteobacteria* (↑) ^d^, *Klebsiella* (↑) ^d^*, Desulfovibrionaceae* (↑) ^d^*, Succinivibrionaceae* (↑) ^d^	*Bifidobacterium* (↓) ^d^	*Tenericutes* (↑) ^d^, *Synergistetes* (↑) ^d^
Picchianti-Diamanti et al. [22]	Italy	*Clostridiaceae* (↓) [*p* = 0.05] ^f^	NA	*Deltaproteobacteria* (↓) [ *p* = 0.05] ^f^, *Enterobacteriales* (↓) [*p* = 0.05] ^c^	NA	*Cyanobacteria* (↑) [*p* = 0.016] ^f^*, Nostocophycidae* (↑) ^f^, *Nostocales* (↑) [*p* = 0.031] ^f^
Scher et al. [17]	USA	NA	*Bacteroides* (↑) ^c^	NA	NA	NA
Chen et al. [19]	USA	*Faecalibacterium* (↑) [*p* < 0.05] ^c^	NA	NA	*Eggerthella lenta* (↑) ^d^, *Collinsella aerofaciens* (↑) ^d^	NA
Muñiz-Pedrogo et al. [34]	USA	*Clostridiaceae* (↑) [*p* = 0.045] ^d^ *Eubacterium* (↑) ^d^	NA	*Epsilonproteobacteria* (↑) [*p* = 0.03] ^d^, *Campylobacteria,* (↑) [*p*=0.04] ^d^	NA	NA
Nayak et al. [25]	USA	NA	*Bacteroidetes* (↓) ^c^	NA	NA	NA
Isaac et al. [37]	USA	*Clostridia* (↑) [*p* < 0.05] ^c^	*Bacteroidia* (↓) [*p* < 0.05] ^c^	NA	NA	NA
Rodrigues et al. [13]	Brazil	*Clostridium leptum* (↓) [*p* = 0.005] ^d^	*Bacteroides* spp. (↑) [*p* = 0.022] ^d^, *Prevotella* spp. (↑) [*p* = 0.023] ^d^	NA	NA	NA
Mena-Vázquez et al. [32]	Spain	*Enterococcus* (↑) [*p* = 0.008] ^d^, *Sedimentibacter* (↑) [*p* = 0.037] ^d^, *Dorea formicigenerans* (↓) [*p* = 0.044] ^d^	NA	NA	*Collinsella aerofaciens* (↑) [*p* = 0.039] ^a^	NA

↑/↓, increased/decreased in patients with rheumatoid arthritis without regimen to treatment. *p*-value < 0.05 for the association in the increased or decreased in the group of the phylum bacteria in the RA population. ^a^ Treatment under therapy with biologics. ^b^ Treatment under therapy with DMARDs + Cs. ^c^ Treatment under therapy with MTX. ^d^ Treatment under therapy with DMARDs + Biologics + Cs. ^e^ Treatment under therapy with SSZ. ^f^ Treatment under therapy with ETN. *p*-value < 0.05 for the association in the increase or decrease in the group of phylum bacteria in the RA population. Legend: NA, not available; RA, rheumatoid arthritis.

**Table 3:** Effect of DMARDs on the gut microbiota and the phenotype to therapy in RA patients.

Author, Year	Country	Time of Study	Therapy Method (*n*)	Responder (*n*)	Non-Responder (*n*)
Neumann et al. [4]	England	0–16 weeks	26 RA (14 SSZ/12 DPA)	NA	NA
Bradley et al. [6]	England	0–16 weeks	31 RA (SSZ)	↓*Clostridium perfringes* in 7 RA patients. ↓IgG antibodies of *Clostridium perfringes*	↑*Clostridium perfringes* in 8 RA patients
Kanerud et al. [102]	Sweden	0–16 weeks	17 RA (SSZ)	NA	NA
Zhang et al. [33]	China	0–12 weeks	34 RA (MTX/T2/MTX+T2)	NA	↑*Lactobacillus salivarus*
Nayak et al. [25]	USA	0–4 weeks	23 RA (MTX)	↓*Bacteroidetes* in 8 RA patients	↑*Bacteroidetes* in 15 RA patients
Isaac et al. [37]	USA	0–16 weeks	27 RA (MTX)	↓Bacterial diversity in 11 RA patients	↑*Clostridia* and ↓*Bacteroidia* in 16 RA patients

↑/↓, increased/decreased in RA patients with a regimen to treatment. Legend: DMARDs, disease-modifying antirheumatic drugs; DPA, D-penicilamine; MTX, methotrexate; NA, not available; RA, rheumatoid arthritis; SSZ, sulfasalazine; T2, Tripterygium wilfordii.
